# Computer-aided molecular design of pyrazolotriazines targeting glycogen synthase kinase 3

**DOI:** 10.1080/14756366.2018.1530223

**Published:** 2018-10-26

**Authors:** M. Lourdes Sciú, Victor Sebastián-Pérez, Loreto Martinez-Gonzalez, Rocio Benitez, Daniel I. Perez, Concepción Pérez, Nuria E. Campillo, Ana Martinez, E. Laura Moyano

**Affiliations:** a Department of Chemical and Physical Biology, Centro de Investigaciones Biológicas (CIB, CSIC) Ramiro de Maeztu, Madrid, Spain;; b INFIQC- Department of Organic Chemistry, School of Chemical Sciences, National University of Córdoba, Córdoba, Argentine;; c Instituto de Química Médica (IQM, CSIC), Madrid, Spain

**Keywords:** GSK-3 inhibitors, pyrazolotriazinones, Alzheimer’s disease, drug design

## Abstract

Numerous studies have highlighted the implications of the glycogen synthase kinase 3 (GSK-3) in several processes associated with Alzheimer’s disease (AD). Therefore, GSK-3 has become a crucial therapeutic target for the treatment of this neurodegenerative disorder. Hereby, we report the design and multistep synthesis of ethyl 4-oxo-pyrazolo[4,3-*d*][1–3]triazine-7-carboxylates and their biological evaluation as GSK-3 inhibitors. Molecular modelling studies allow us to develop this new scaffold optimising the chemical structure. Potential binding mode determination in the enzyme and the analysis of the key features in the catalytic site are also described. Furthermore, the ability of pyrazolotriazinones to cross the blood–brain barrier (BBB) was evaluated by passive diffusion and those who showed great GSK-3 inhibition and permeation to the central nervous system (CNS) showed neuroprotective properties against tau hyperphosphorylation in a cell-based model. These new brain permeable pyrazolotriazinones may be used for key *in vivo* studies and may be considered as new leads for further optimisation for the treatment of AD.

## 1. Introduction

Alzheimer’s disease (AD) is a neurodegenerative disorder characterised by memory loss and progressive impairment in cognitive functions. To date, no cure has been found that stops or reverses the underlying progression of the disease^1^. Although the cause of AD remains unknown, three main hypotheses have been developed that define the neuropathological profile of this disease. AD is characterised by three key structural changes in the brain such as neuronal loss, extracellular β-amyloid plaques accumulations, and intracellular neurofibrillary tangles (NFT) deposits formation by hyperphosphorylation of tau protein^2^.

Protein kinases are key regulator of cellular functions since they direct the activity, localisation, and overall function of many proteins by adding phosphate groups to protein substrates. As protein kinases are involved in the regulation of almost every cellular process, they have become the main target of study for the treatment of several diseases. The glycogen synthase kinase 3 (GSK-3) is a serine/threonine kinase involved in the regulation of glycogen synthesis and modulation of other intracellular processes. Over-activation of GSK-3 is implicated in several processes associated with AD, such as tau hyperphosphorylation, increase of β-amyloid plaques production, microglia activation, neurofibrillary tangle formation, and cell apoptosis^3^^,^^4^.

Moreover, a full etiopathological hypothesis for AD has been proposed based on GSK-3 dysfunction^5^^,^^6^. Therefore, this kinase is one of the main targets studied for the treatment of the neurodegenerative disease. Over the last few years, different heterocyclic compounds have been described as GSK-3 inhibitors including natural products as manzamine alkaloids^7^ and palinurine^8^, but a great number of chemical substances come from synthetic approaches. Among them, pyrazol-5-ones^9^, pyrazolo-fused derivatives^10–14^, pyrazines^15^, thiadiazolidinones^16^^,^^17^, triazinones^18^, are good examples. Some of them contain privileged scaffolds, as the pyrazole nucleus, and others can be classified as purine analogues, since they are structurally related to purine due to their fused five and six-membered nitrogen heterocycles^19^.

Purine analogues have been widely studied in drug design due to the action of purine nucleotides like ATP, GTP, cAMP, cGMP, NAD, FAD, as co-factors, substrates or mediators in the functioning of many proteins^19^^,^^20^. In fact, structurally purine related compounds have shown multiple biological activities, such as antiviral, antibacterial, anti-inflammatory activity, among others^20–22^. Following our efforts to attain purine analogues of biological interest, hereby we describe the evaluation of pyrazolo[3,4-*d*][1–3]triazin-4-one as potential GSK-3 inhibitors and docking studies to determine the binding mode to the enzyme. Subsequently, molecular modelling was used to guide the chemical optimisation of these compounds. New ethyl 4-oxo-pyrazolo[4,3-*d*][1–3]triazine-7-carboxylates were conceived by computational tools, synthesised from accessible precursors and evaluated as GSK-3 inhibitors. Their ability to cross the blood–brain barrier by passive diffusion was also evaluated to consider them as potential drug candidates for the treatment of AD.

## 2. Materials and methods

### 2.1. Molecular modelling

#### 2.1.1. Protein preparation

The protein 4NM5^23^ was retrieved from the protein data bank and was prepared using Protein Preparation Wizard of Schrodinger Suite^24^. 4NM5 was selected because it is crystallized with a purine derivative, a similar scaffold to the compounds described here. Also, present a good resolution and present no gaps comparing with other structures. The protein structure was checked to assign correct bond orders, hydrogen atoms were added, and waters were deleted beyond 5 Å of heteroatoms groups. The hydrogen bonds were optimised and assigned and the protonation states of all the residues were optimised using a physiological pH of 7.3. All the waters molecules were removed and finally, an energy restrained minimisation was carried out using default constraint of 0.3 Å RMSD and OPLS 2005 force field^25^.

#### 2.1.2. Docking

Automated docking was used to assess the appropriate binding orientations and conformations of the ligand molecules with different protein inhibitors. A Lamarckian genetic algorithm method implemented in the programme AutoDock 4.2 was employed^26^^,^^27^; to carry out the docking simulations AutoDock suite was used. For docking calculations, Gasteiger charges were added and the rotatable bonds were set by AutoDock tools (ADT) and all torsions could rotate for the ligand. The ligand was treated as a flexible molecule while the protein was treated as rigid. Polar hydrogen atoms were added and Gasteiger charges were assigned to the protein, using ADT and AD4 atom type was assigned. The modified structures obtained were converted in PDBQT format in ADT for AutoDock calculations.

#### 2.1.3. Validation

To validate the docking protocol, 1Q3W was retrieved from pdb. This pdb was selected because it contains alsterpaullone, one of the most potent known inhibitors of GSK3. Alsterpaullone and pdb protein structure were prepared as described above. Grid was centred in Val135 using a grid box size of 50 × 50 × 50 points with a grid-point spacing of 0.375 Å. The grid maps were generated by Autogrid programme.

#### 2.1.4. Evaluation

For the compounds evaluated the grid was centred in Val135, as it has been proved to be an important residue in the protein-ligand recognition. The grid maps were generated by Autogrid programme. In all the cases grid maps with a grid box size of 50 × 50 × 50 Å^3^ and a grid-point spacing of 0.375 Å were used. The docking protocol for all the compounds consisted of 200 independent Genetic Algorithm (GA) runs per ligand, population size of 150, maximum number of evaluation 2,500,000, maximum number of 27,000 generation, mutation rate of 0.02 and a crossover rate of 0.8 were used for this study. The docking results for a given macromolecule-ligand pair mainly comprised of the intermolecular interaction energies including inhibition constant, hydrogen bond interaction energy, van der Waals forces, electrostatic energy and ligand efficiency. Finally, best-docked clusters (within the default 2.0 Å RMSD) according to the binding energies and relative population provided by Autodock were analysed by visual inspection.

Analysis of the receptor-ligand complex models was based on energetic score, population of the conformations in the different clusters and interactions. Interactions considered were hydrogen bonds, aromatic and hydrophobic ones, all were predicted with Maestro.

#### 2.1.5. Hotspot analysis

After preparation of the protein as described above, the hotspot maps were calculated using the protocol developed by the group of Prof. Blundell^28^. To generate fragment hotspots, atomic hotspots are first calculated. SuperStar calculated the atomic hotspots. Three maps were generated: hydrophobic − aromatic CH probe, donor − uncharged NH probe, acceptor − carbonyl O probe. SuperStar uses the LIGSITE32 algorithm to detect cavities, and in the absence of a starting coordinate or residue from which to grow the cavity, LIGSITE runs on the whole protein.

It gives each grid point a buriedness score between zero (completely solvent exposed) and seven (completely buried). SuperStar then provided atomic propensities for cavities that contained grid points with a LIGSITE score of five or above. To find areas where high interaction propensity coincides with buried pockets, the SuperStar maps are weighted by the LIGSITE score for each grid point. The weighted SuperStar maps show propensity throughout the protein, but in order to find fragment hotspots, the atomic propensities were sampled with molecular probes. These probes were toluene for a polar region, aniline for donors region and a carbonyl for acceptors region.

### 2.2. Chemistry

#### 2.2.1. General chemical methods

All reagents were obtained from commercial sources and used without further purification. Proton (^1^H) and Carbon (^13^C) NMR spectra were recorded on a 400 MHz Bruker spectrometer (^1^H at 400.16 MHz and ^13 ^C at 100.9 MHz) at ambient temperature. Solutions were typically prepared in either deuterochloroform (CDCl_3_), deuteroacetone ((CD_3_)_2_CO), deuterated acetonitrile (CD_3_CN) or deuterated dimethylsulphoxide ((CD_3_)_2_SO) with chemical shifts referenced to deuterated solvent as an internal standard. ^1^H NMR data are reported indicating the chemical shift (δ), the multiplicity (s, singlet; d, doublet; t, triplet; q, quartette; m, multiplet; br, broad; dd, doublet of doublets, etc.), the coupling constant (*J*) in Hz and the integration (e.g. 1H). High-resolution mass spectra (HRMS) were recorded on a LC-MS Bruker Daltonics Micro TOFF QII with electrospray ionisation (ESI) and Q-TOF detection.

#### 2.2.2. General procedure for the synthesis of N-aryl-hydrazones (7a–h)

To an ice-cold solution of the aniline **6** (1.0 mmol) and 37% aq. HCl (1.1 ml, 13.4 mmol) in water (5 ml mmol^−1^) was added dropwise 1 M aq. NaNO_2_ (1 ml, 1.0 mmol). The mixture was stirred for 30 min and then added dropwise to a solution of malononitrile (0.09 ml, 1.5 mmol) and sodium acetate trihydrate (4.22 g, 31.0 mmol) in water (8.5 ml mmol^−1^ of aniline) with continuous stirring and cooling to 0 °C. After 2 h, the insoluble hydrazone was isolated by filtration and washed with cold water. The desired hydrazone was used without further purification in the next step.

#### 2.2.3. General procedure for the preparation of ethyl 4-amino-3-cyano-1H-pyrazole-5-carboxylates (8a–h)

A mixture of hydrazone **7** (1.0 mmol), potassium carbonate (0.138 g, 1.0 mmol), and ethyl bromoacetate (0.45 g, 2.7 mmol) in dimethylformamide (3 ml mmol^−1^) was heated at 90 °C for 5 to 7 h. After the reaction was finished, the mixture was poured in a mix of ice and water and kept at 4 °C for 4–5 h. The residue obtained was isolated by filtration and washed with cold water. The resulting solid was used without further purification for compounds **8a**–**c** and **8g**. Instead, the solid obtained for compounds **8d**–**f** and **8h** was recrystallized from ethanol.

Ethyl 4-amino-3-cyano-1-phenyl-1*H*-pyrazole-5-carboxylate (**8a**): This compound [CAS 58838–10-1] was obtained as a pale orange solid in 39% yield, m.p. 113–116 °C. ^1^H NMR (400 MHz, CD_3_CN, 25 °C): δ = 7.56–7.33 (m, 5H), 5.15 (s, 2H), 4.18 (q, *J*  =  7.1 Hz, 2H), 1.11 (t, *J*  =  7.1 Hz, 3H) ppm. ^13 ^C NMR (101 MHz, (CD_3_)_2_CO, 25 °C): δ = 159.7, 143.8, 141.5, 129.4 (2C), 126.9 (2C), 118.1, 114.7, 113.5, 61.5, 14.2 ppm.

Ethyl 4-amino-3-cyano-1-(*p*-tolyl)-1*H*-pyrazole-5-carboxylate (**8b**): This compound [CAS 1237748–26-3] was obtained as an orange solid in 38% yield, m.p. 122–124 °C. ^1^H NMR (400 MHz, (CD_3_)_2_CO, 25 °C): δ = 7.42–7.23 (m, 4H), 5.65 (s, 2H), 4.21 (q, *J*  =  7.1 Hz, 2H), 2.41 (s, 3H), 1.14 (t, *J*  =  7.1 Hz, 3H) ppm. ^13 ^C NMR (101 MHz, CD_3_CN, 25 °C): δ = 159.9, 143.4, 140.5, 139.0, 131.1, 130.0 (2C), 126.8 (2C), 114.5, 113.8, 61.9, 21.23 14.3 ppm.

Ethyl 4-amino-3-cyano-1–(4-methoxyphenyl)-1*H*-pyrazole-5-carboxylate (**8c**): This compound was obtained as an orange solid in 42% yield, m.p. 124–126 °C. ^1^H NMR (400 MHz, (CD_3_)_2_CO, 25 °C): δ = 7.45–7.37 (m, 2H), 7.07–7.01 (m, 2H), 5.62 (s, 2H), 4.21 (q, *J*  =  7.1 Hz, 2H), 3.88 (s, 3H), 1.14 (t, *J*  =  7.1 Hz, 3H) ppm. ^13 ^C NMR (101 MHz, CD_3_CN, 25 °C): δ = 161.2, 159.9, 143.4, 134.5, 128.3 (2C), 115.9, 114.6 (2C), 114.6, 114.3, 113.9, 61.9, 56.4, 14.3 ppm. IR (AgBr): υ = 3471, 3359 (NH), 2230 (CN), 1721 (C=O), 1617, 1514 (C=C and C=N ring) cm^−1^. HRMS calculated for C_14_H_14_N_4_O_3_H^+^: 287.11387, found: 287.11996.

Ethyl 4-amino-3-cyano-1–(4-fluorophenyl)-1*H*-pyrazole-5-carboxylate (**8d**): This compound was obtained as a pale brown solid in 49% yield, m.p. 118–124 °C. ^1^H NMR (400 MHz, CDCl_3_, 25 °C): δ = 7.42–7.30 (m, 2H), 7.21–7.10 (m, 2H), 4.80 (s, 2H), 4.23 (q, *J*  =  7.1 Hz, 2H), 1.17 (t, *J*  =  7.1 Hz, 3H) ppm. ^13 ^C NMR (101 MHz, (CD_3_)_2_CO), 25 °C) δ = 163.6 (d, *J*  =  246.9 Hz), 159.6, 143.7, 137.8, 129.2 (d, *J*  =  9.1 Hz, 2C), 118.3, 116.1 (d, *J*  =  23.4 Hz, 2C), 114.7, 113.4, 61.6, 14.2 ppm. IR (AgBr): υ = 3475, 3369 (NH), 2229 (CN), 1721 (C=O), 1616, 1511 (C=C and C=N ring) cm^−1^. HRMS calculated for C_13_H_11_FN_4_O_2_H^+^: 275.09388, found: 275.09379.

Ethyl 4-amino-1–(4-chlorophenyl)-3-cyano-1*H*-pyrazole-5-carboxylate (**8e**): This compound was obtained as brown solid in a 52% yield, m.p. 128–133 °C. ^1^H NMR (400 MHz, CD_3_CN, 25 °C): δ = 7.49 (d, *J*  =  8.8 Hz, 2H), 7.41 (d, *J*  =  8.8 Hz, 2H), 5.17 (s, 2H), 4.20 (q, *J*  =  7.1 Hz, 2H), 1.14 (t, *J*  =  7.1 Hz, 3H) ppm. ^13 ^C NMR (101 MHz, CD_3_CN, 25 °C): δ = 159.8, 143.6, 140.1, 135.5, 129.6 (2C), 128.8, 128.6 (2C), 115.1, 113.6, 62.1, 14.3 ppm. IR (AgBr): υ = 3424, 3309 (NH), 2230 (CN), 1724 (C=O), 1627, 1498 (C=C and C=N ring) cm^−1^. HRMS calculated for C_13_H_11_ClN_4_O_2_H^+^: 291.06433, found: 291.06441.

Ethyl 4-amino-1–(4-bromophenyl)-3-cyano-1*H*-pyrazole-5-carboxylate (**8f**): This compound was obtained as a brown solid in a 40% yield, m.p. 132–134 °C. ^1^H NMR (400 MHz, CDCl_3_, 25 °C): δ = 7.59 (d, *J*  =  8.7 Hz, 2H), 7.27 (d, *J*  =  8.7 Hz, 2H), 4.81 (s, 2H), 4.26 (q, *J*  =  7.1 Hz, 2H), 1.20 (t, *J*  =  7.1 Hz, 3H) ppm. ^13 ^C NMR (101 MHz, CD_3_CN, 25 °C): δ = 159.8, 143.6, 140.6, 133.2, 132.7 (2C), 128.9 (2C), 123.5, 120.5, 113.6, 62.1, 14.3 ppm. IR (AgBr): υ = 3426, 3309 (NH), 2230 (CN), 1723 (C=O), 1627, 1490 (C=C and C=N ring) cm^−1^. HRMS calculated for C_13_H_11_BrN_4_O_2_H^+^: 335.01381, found: 335.01368.

Ethyl 4-amino-3-cyano-1–(4-iodophenyl)-1*H*-pyrazole-5-carboxylate (**8g**): This compound was obtained as brown solid in a 55% yield, m.p. 135–138 °C. ^1^H NMR (400 MHz, (CD_3_)_2_CO, 25 °C): δ = 7.95–7.86 (m, 2H), 7.37–7.31 (m, 2H), 5.70 (s, 2H), 4.24 (q, *J*  =  7.1 Hz, 2H), 1.17 (t, *J*  =  7.1 Hz, 3H) ppm. ^13 ^C NMR (101 MHz, CD_3_CN, 25 °C): δ = 159.8, 143.6, 141.2, 139.4, 138.7 (2C), 128.9 (2C), 115.2, 113.6, 95.0, 62.1, 14.3 ppm. IR (AgBr): υ = 3450, 3361 (NH), 2233 (CN), 1700 (C=O), 1640, 1489 (C=C and C=N ring) cm^−1^. HRMS calculated for C_13_H_11_IN_4_O_2_H^+^: 382.99995, found: 382.999978.

Ethyl 4-amino-3-cyano-1–(4-nitrophenyl)-1*H*-pyrazole-5-carboxylate (**8h**): This compound was obtained as an orange solid in a 18% yield. ^1^H NMR (400 MHz, (CD_3_)_2_SO, 25 °C): δ = 8.33 (d, *J*  =  8.8 Hz, 2H), 7.81 (d, *J*  =  8.8 Hz, 2H), 6.16 (s, 2H), 4.19 (q, *J*  =  6.9 Hz, 2H), 1.13 (t, *J*  =  7.1 Hz, 3H) ppm. ^13 ^C NMR (101 MHz, CD_3_CN, 25 °C): δ = 159.8, 148.8, 145.8, 143.9, 127.9 (2C), 125.9, 125.0 (2C), 116.3, 113.4, 62.3, 14.3 ppm.

#### 2.2.4. General procedure for the synthesis of 4-oxo-4,6-dihydro-3H-pyrazolo[4,3-d][1–3]triazine-7-carboxylates (5a–c,e–g)

Aqueous NaNO_2_ (1 ml, 1.8 mmol) was added to a stirred and cooled solution (0–5 °C) of pyrazole **8** (1.0 mmol) in a mixture of HCl: AcOH (3:1, 20 ml) over a period of 10 min. The reaction mixture was allowed to warm at room temperature and was stirred for 20 h. The precipitate of ethyl-pyrazolotriazine-carboxylate obtained was filtered off and then diluted with water (20 ml). After that, the solution was extracted with dichloromethane (3 × 20 ml) and the organic phase was dried with anhydrous MgSO_4_. The resulting solution was concentrated to dryness, and the solid was then subject to chromatographic column separation with dichloromethane and dichloromethane/ethyl acetate in different proportions.

Ethyl 4-oxo-6-phenyl-4,6-dihydro-3*H*-pyrazolo[4,3-*d*][1–3]triazine-7-carboxylate (**5a**): This compound was obtained as a pale yellow solid in a 35% yield, m.p. 191.5–193 °C. ^1^H NMR (400 MHz, (CD_3_)_2_CO, 25 °C): δ = 13.90 (s, 1H), 7.77–7.54 (m, 5H), 4.38 (q, *J*  =  7.1 Hz, 2H), 1.27 (t, *J*  =  7.1 Hz, 3H) ppm. ^13 ^C NMR (100 MHz, (CD_3_)_2_CO, 25 °C): δ = 158.2, 152.9, 140.8, 137.4, 135.6, 131.0, 130.6, 129.9 (2C), 126.8 (2C), 63.0, 14.2 ppm. IR (AgBr): υ = 1723 (C=O), 1496 (C=N ring) cm^−1^. HRMS calculated for C_13_H_11_N_5_O_3_Na^+^: 308.0754; found: 308.0760.

Ethyl 4-oxo-6-(p-tolyl)-4,6-dihydro-3*H*-pyrazolo[4,3-*d*][1–3]triazine-7-carboxylate (**5b**): This compound was obtained as a yellow solid in 38% yield, m.p. 193–196 °C. ^1^H NMR (400 MHz, (CD_3_)_2_SO, 25 °C): δ = 14.99 (s, 1H), 7.52 (d, *J*  =  8.4 Hz, 2H), 7.39 (d, *J*  =  8.1 Hz, 2H), 4.33 (q, *J*  =  7.1 Hz, 2H), 2.43 (s, 3H), 1.21 (t, *J*  =  7.1 Hz, 3H) ppm. ^13 ^C NMR (100 MHz, (CD_3_)_2_SO, 25 °C): δ = 157.1, 152.3, 140.1, 137.0, 136.1, 134.0, 129.4 (2C), 128.8, 125.7 (2C), 62.1, 20.8, 13.8 ppm. IR (AgBr): υ = 1706, 1725 (C=O), 1511 (C=N ring) cm^−1^. HRMS calculated for C_14_H_13_N_5_O_3_Na^+^: 322.0911; found: 322.0906.

Ethyl 6–(4-methoxyphenyl)-4-oxo-4,6-dihydro-3*H*-pyrazolo[4,3-*d*][1–3]triazine-7-carboxylate (**5c**): This compound was obtained as a light brown solid in 28% yield, m.p. 194 °C (dec.). ^1^H NMR (400 MHz, CDCl_3_, 25 °C): δ = 11.55 (s, 1H), 7.47 (d, *J*  =  8.9 Hz, 2H), 7.03 (d, *J*  =  8.9 Hz, 2H), 4.47 (q, *J*  =  7.1 Hz, 2H), 3.90 (s, 3H), 1.37 (t, *J*  =  7.1 Hz, 3H) ppm. ^13 ^C NMR (101 MHz, CDCl_3_, 25 °C): δ = 161.2, 158.8, 157.7, 151.9, 134.4, 132.2, 127.1 (2C), 114.3 (2C), 109.9, 63.0, 55.8, 29.9 ppm. IR (AgBr): υ = 1714, 1725 (C=O), 1512 (C=N ring) cm^−1^. HRMS calculated for C_14_H_13_N_5_O_4_Na^+^: 338.0806; found: 338.0863.

Ethyl 6–(4-chlorophenyl)-4-oxo-4,6-dihydro-3*H*-pyrazolo[4,3-*d*][1–3] triazine-7-carboxylate (**5e**): This compound was obtained as a pale yellow solid in a 31% yield, m.p. 198 °C (dec.). ^1^H NMR (400 MHz, CD_3_CN, 25 °C): δ = 12.70 (s, 1H), 7.59 (m, 4H), 4.38 (q, *J*  =  7.1 Hz, 2H), 1.26 (t, *J*  =  7.1 Hz, 3H) ppm. ^13 ^C NMR (101 MHz, CD_3_CN, 25 °C): δ = 158.3, 153.1, 139.4, 137.6, 136.7, 135.8, 130.8, 130.1 (2C), 128.7 (2C), 63.6, 14.2 ppm. IR (AgBr): υ = 1723, 1710 (C=O), 1496 (C=N ring) cm^−1^. HRMS calculated for C_13_H_10_ClN_5_O_3_Na^+^: 342.0364; found: 342.0374.

Ethyl 6–(4-bromophenyl)-4-oxo-4,6-dihydro-3*H*-pyrazolo[4,3-*d*][1–3]triazine-7-carboxylate (**5f**): This compound was obtained as a yellow solid in 36% yield, m.p. 195 °C (dec.). ^1^H NMR (400 MHz, (CD_3_)_2_CO, 25 °C): δ = 13.93 (s, 1H), 7.83 (d, *J*  =  8.8 Hz, 2H), 7.67 (d, *J*  =  8.8 Hz, 2H), 4.41 (q, *J*  =  7.1 Hz, 2H), 1.30 (t, *J*  =  7.1 Hz, 3H) ppm. ^13 ^C NMR (101 MHz, (CD_3_)_2_CO, 25 °C): δ = 158.2, 152.8, 140.0, 137.4, 135.8, 133.0 (2C), 130.6, 129.0 (2C), 124.6, 63.1, 14.3 ppm. IR (AgBr): υ = 1710 broad signal (C=O), 1490 (C=N ring) cm^−1^. HRMS calculated for C_13_H_10_BrN_5_O_3_Na^+^: 385.9859; found: 385.9868.

Ethyl 6–(4-iodophenyl)-4-oxo-4,6-dihydro-3*H*-pyrazolo[4,3-*d*][1–3]triazine-7-carboxylate (**5g**): This compound was obtained as a yellow solid in 33% yield. m.p. 198 °C (dec.). ^1^H NMR (400 MHz, (CD_3_)_2_CO, 25 °C): δ = 13.90 (s, 1H), 7.77–7.54 (m, 4H), 4.38 (q, *J*  =  7.1 Hz, 2H), 1.27 (t, *J*  =  7.1 Hz, 3H) ppm. ^13 ^C NMR (101 MHz, (CD_3_)_2_CO, 25 °C): δ = 158.2, 152.8, 140.7, 139.1 (2C), 137.4, 135.8, 130.6, 128.9 (2C), 96.3, 63.1, 14.3 ppm. IR (AgBr): υ = 1723 broad signal (C=O), 1490 (C=N ring) cm^−1^. HRMS calculated for C_13_H_10_IN_5_O_3_Na^+^: 433.9721; found: 433.9710.

### 2.3. Biology

#### 2.3.1. Inhibition of GSK-3β

Human recombinant GSK-3β and the pre-phosphorylated polypeptide substrate were purchased from Millipore (Millipore Ibérica SAU). Kinase-Glo Luminescent Kinase Assay was obtained from Promega (Promega Biotech Ibérica, SL)^29^. ATP and all other reagents were from Sigma Aldrich (St. Louis, MO). Assay buffer contained 50 mM HEPES (pH 7.5), 1 mM EDTA, 1 mM EGTA, and 15 mM magnesium acetate.

The method of Baki et al. was followed to analyse the inhibition of GSK-3β^30^. Kinase-Glo assays were performed in assay buffer using black 96-well plates. In a typical assay, 10 μL (10 μM) of test compound (dissolved in dimethyl sulfoxide [DMSO] at 1 mM concentration and diluted in advance in assay buffer to the desired concentration) and 10 μL (20 ng) of enzyme were added to each well followed by 20 μL of assay buffer containing 25 μM substrate and 1 μM ATP. The final DMSO concentration in the reaction mixture did not exceed 1%. After 30 min incubation at 30 °C, the enzymatic reaction was stopped with 40 μL of Kinase-Glo reagent. Glow-type luminescence was recorded after 10 min using a FLUOstar Optima (BMG Labtechnologies GmbH, Offenburg, Germany) multimode reader. The activity is proportional to the difference of the total and consumed ATP. The inhibitory activities were calculated based on maximal activities measured in the absence of inhibitor. The IC_50_ was defined as the concentration of each compound that reduces a 50% the enzymatic activity with respect to that without inhibitors.

#### 2.3.2. Kinetic studies on GSK-3β

To investigate the inhibitory mechanism of compounds **5** on GSK-3β, several kinetic experiments were performed using the ADP-Glo Kinase Assay. Kinetic experiments varying both ATP (from 1 to 50 μM) and the inhibitor (at 0.5 and 1 μM) concentrations were performed, while the concentration of GS-2 was kept constant at 12.5 μM.

#### 2.3.3. *In vitro* parallel artificial membrane permeability assay (PAMPA)

Prediction of the brain penetration was evaluated using a parallel artificial membrane permeability assay (PAMPA). Ten commercial drugs, phosphate buffer saline solution at pH 7.4 (PBS), ethanol, and dodecane were purchased from Sigma Aldrich, Across organics and Fluka. The porcine polar brain lipid (PBL) (catalogue no. 141101) was from Avanti Polar Lipids. The donor plate was a 96-well filtrate plate (Multiscreen IP Sterile Plate PDVF membrane, pore size is 0.45 μM, catalogue no. MAIPS4510), and the acceptor plate was an indented 96-well plate (Multiscreen, catalogue no. MAMCS9610), both from Millipore. Filter PDVF membrane units (diameter 30 mm, pore size 0.45 μm) from Symta were used to filter the samples. A 96-well plate UV, (Thermoscientific, Varioskan Lux multimode microplate reader) was used for the UV measurements. Test compounds [(3−5 mg of caffeine, enoxacine, hydrocortisone, desipramine, ofloxacine, piroxicam, and testosterone), (12 mg of promazine), and 25 mg of verapamil and atenolol] were dissolved in ethanol (1000 μL). Then 100 μL of this compound stock solution was taken, and 1400 μL of ethanol and 3500 μL of PBS pH 7.4 buffer were added to reach 30% of ethanol concentration in the experiment. These solutions were filtered. The acceptor 96-well microplate was filled with 180 μL of PBS:ethanol (70:30). The donor 96-well plate was coated with 4 μL of porcine brain lipid in dodecane (20 mg mL^−1^), and after 5 min, 180 μL of each compound solution was added. Then 1−2 mg of every compound to be determined for their ability to pass the brain barrier were dissolved in 1500 μL of ethanol and 3500 μL of PBS pH 7.4 buffer, filtered, and then added to the donor 96-well plate. Then the donor plate was carefully put on the acceptor plate to form a “sandwich”, which was left undisturbed for 4 h at 25 °C. During this time, the compounds diffused from the donor plate through the brain lipid membrane into the acceptor plate. After incubation, the donor plate was removed. The concentration of compounds and commercial drugs in the acceptor and the donor wells was determined by UV plate reader. Every sample was analysed at three to five wavelengths, in three wells and in three independent runs. Results are given as the mean [standard deviation (SD)], and the average of the three runs is reported. Ten quality control compounds (previously mentioned) of known BBB permeability were included in each experiment to validate the analysis set.

#### 2.3.4. Okadaic acid-induced tau hyperphosphorylation cell model

Human SH-SY5Y cells were grown in DMEM supplemented with 10% FBS and 1% penicillin/streptomycin at 37 °C and 5% CO_2_ in an incubator. SH-SY5Y cells were seeded onto 96-well plate at 60.000 cells per well. 48 h later, cells were pre-incubated with the compounds at the desired concentration for 1 h and after that time okadaic acid (OA) (Sigma Aldrich, catalogue no: 09381) was added at a concentration of 30 nM and incubated for another 24 h. Afterwards, cells were incubated with 0.5 mg mL^−1^ MTT solution for at least 4 h at 37 °C and 5% CO_2._ Then culture media was removed and the formazan crystals attached to the bottom of the plate were dissolved with 200 µL of DMSO. Finally, UV-absorbance was measured at 595 nM in a microplate reader (Varioskan Flash Microplate reader, Thermo Scientific).

## 3. Results and discussion

**Scheme 1.**
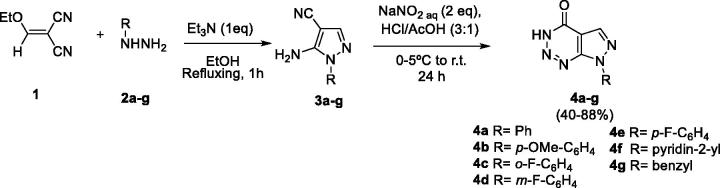
Synthesis of pyrazolo[3,4-*d*][1–3]triazin-4-ones **4a–g**.

Following with our efforts to develop effective and selective of GSK-3 inhibitors, we assayed the potential inhibitory effect of a family of pyrazolo[3,4-*d*][1–3]triazin-4-ones **4** previously synthesised in our group (Scheme 1)^31^.

**Figure 1.**
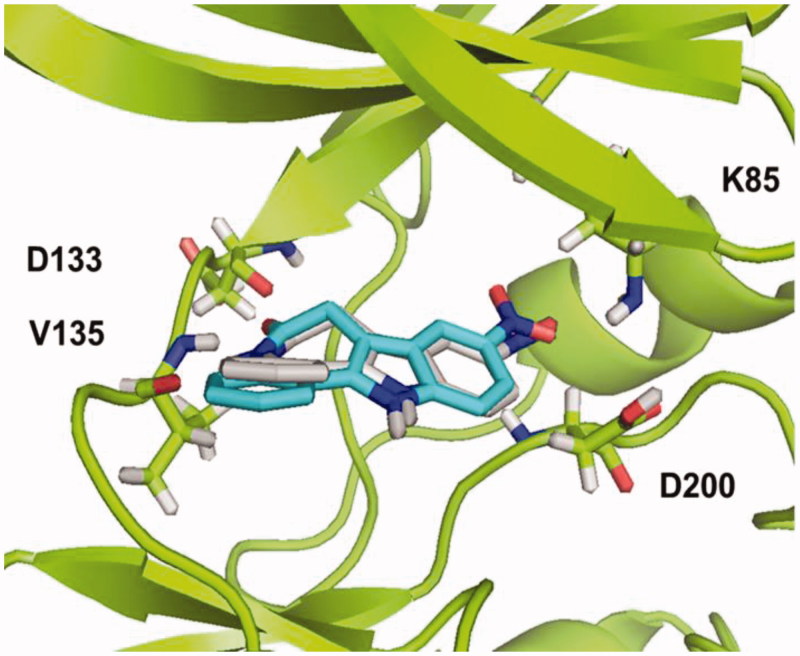
Superimposition of the crystal structure of Alsterpaullone ligand in complex with GSK-3, 1Q3W (blue) and the best pose resulting from docking studies (grey).

This family did not show any GSK-3 inhibition at a fixed compound concentration of 10 μM. To redesign this heterocyclic family of compounds, docking studies were performed to identify structural clues involved in the kinase-small molecule binding. First, a validation protocol was carried out using alsterpaullone as a reference inhibitor^32^. This inhibitor has been crystallized in a complex with the enzyme and the 3D-coordinates are available in the protein data bank 1Q3W. To validate the docking protocol here used, we performed docking studies of the ligand in the ATP-binding site and analysed the best poses found by the programme. Comparing the position of alsterpaullone in the available crystal structure and the docking poses found (Figure 1), it can be concluded that both conformations are very similar, and the interactions found in both cases are the same. These results support the docking protocol developed here.

Once the protocol was validated, docking studies for compounds **4a**–**g** were performed. The results were analysed based on the binding energy, the population of the conformations in each cluster and the main interactions between the pyrazolotriazinones and the enzyme. Both, electrostatic and hydrophobic interactions were analysed.

**Figure 2.**
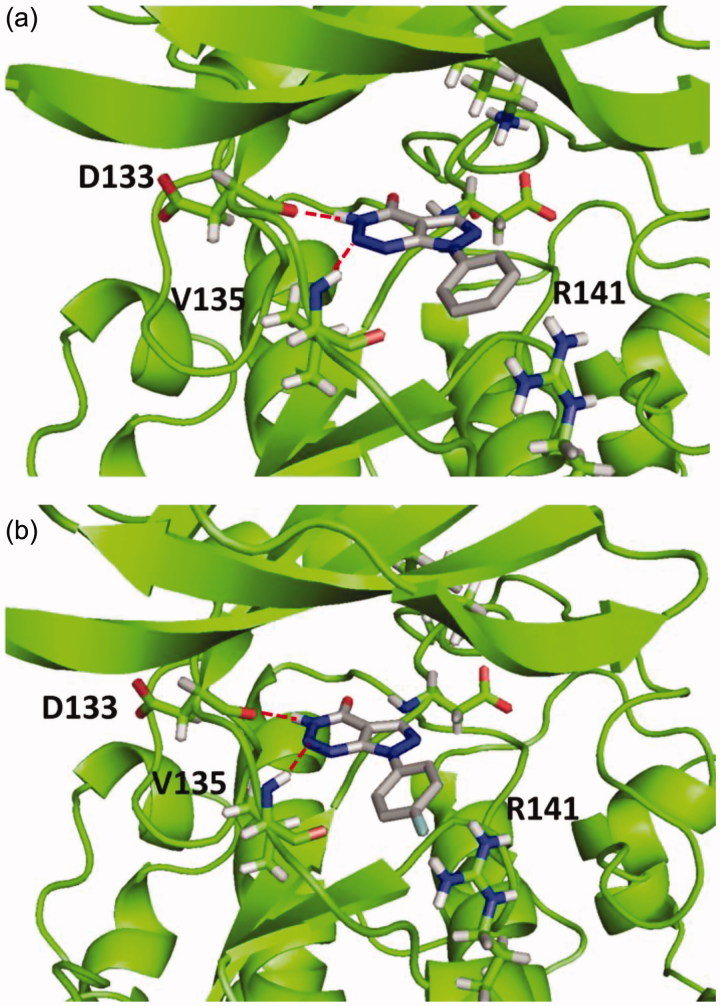
Binding mode for inactive compound **4a** (a) and **4e** (b) in GSK-3 showing two relevant H-bonds with nearby residues Asp133 and Val135 and negative interaction with Arg141, due to steric impediment.

According to the results, all compounds showed a similar binding mode in the catalytic region of GSK-3. As an example, the docking pose obtained for compound **4a** is shown in Figure 2(a). Among the main interactions analysed, two hydrogen bonds were observed between the triazin-4-one moiety and residues Asp133 and Val135. Also, a negative interaction was found because of the orientation of the phenyl ring towards the residue Arg141 due to steric impediment. Moreover, this negative interaction was increased for compounds with a substituent in the *para* position of the phenyl group, such as compound **4e**, as it is shown in Figure 2(b). This interaction could explain why compounds **4a**–**g** turned out to be inactive in GSK-3.

Following, different molecular modelling studies were performed to analyse the enzyme cavity and design new compounds for the potential inhibition of GSK-3.

To study the enzyme cavity, a hotspot analysis was performed^28^. This method samples atomic hotspots with simple molecular probes to produce fragment hotspot maps. These maps specifically highlight fragment-binding sites and their corresponding pharmacophores. For ligand-bound structures, they provide an intuitive visual guide within the binding site, directing medicinal chemists where to make modifications in the molecule to improve potency.

The analysis generates protein hotspots, which are regions within enzyme pockets that might be critical to contribute to the binding mode of the ligand. Identification of hotspots and their specific interactions can be used to evaluate the ligand ability of a pocket and suggest which interactions fragments and larger ligands will be needed to make a stronger binding.

With this study, a colour map of the main interactions can be obtained that could be important for the activity in the enzyme. In this sense, we can identify either novel inhibitors or explain the differences in activities between ligands considering critical protein-ligand recognition features.

**Figure 3.**
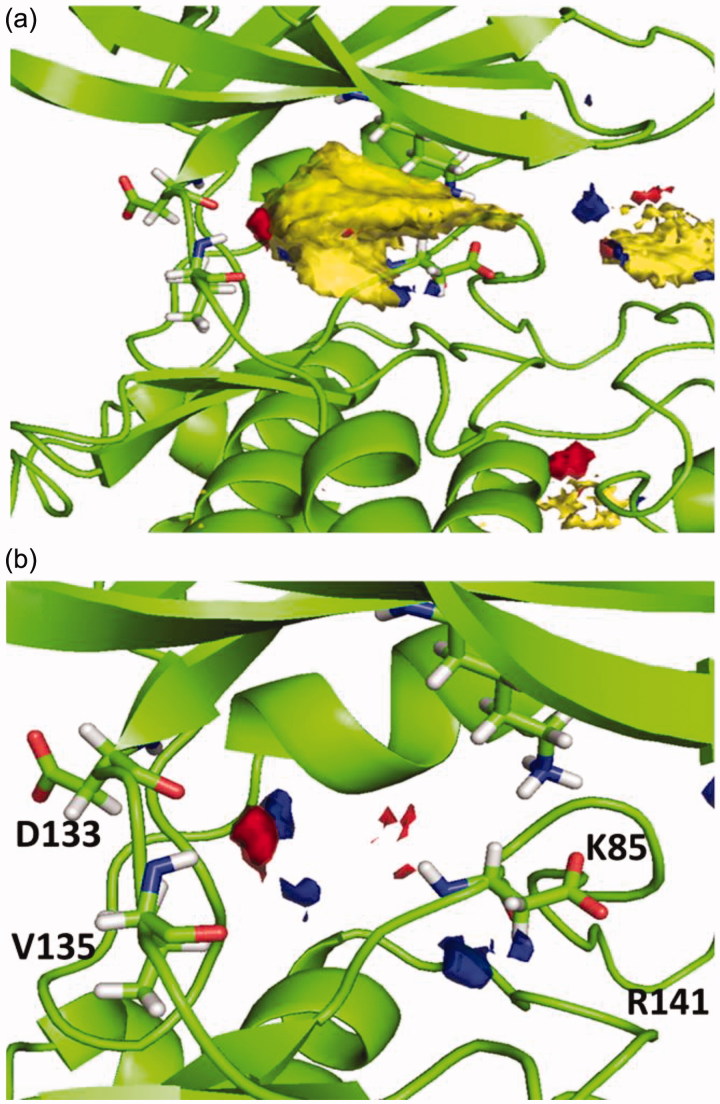
(a) Hotspot analysis of the binding site of the enzyme. (b) Polar regions in the protein cavity that could act as H-bond donor (blue) or H-bond acceptor (red).

Because of the analysis, a surfaces map, shown in Figure 3(a), was obtained, where optimal interactions could be determined depending on the different colours. Thus, the yellow region indicates favourable hydrophobic interactions (Figure 3(a)), while red and blue indicate favourable polar interactions as shown in Figure 3(b).

Considering this analysis, the two hydrogen bonds between **4a** and nearby residues Asp133 and Val135 in the binding site seems relevant, since they match with the hotspot maps. Also, phenyl ring should be removed from the molecule in position N7 to avoid negative interactions and a polar group might be added in this position capable of interact favourable with Arg141. This modification might allow a hydrogen-bond acceptor in the molecule with the Arg141. Moreover, phenyl ring could be re-oriented in the molecule towards the hydrophobic region in the pocket found by the hotspots studies that end near residues Lys85 and Asp200.

Bearing in mind experimental and docking studies of inactive compounds **4a**–**g** together with the hotspot analysis; new GSK-3 potential inhibitors were designed. Since triazin-4-one scaffold interacts favourable with the enzyme cavity, this moiety remains unaltered. Instead, we decided to modify the type of ring fused between the pyrazole and triazine rings, to direct the phenyl substituent towards the hydrophobic region and try to occupy the entire catalytic pocket. Also, a carboethoxy substituent was introduced in the pyrazole ring as a polar group to attempt a favourable interaction with Arg141.

**Figure 4.**
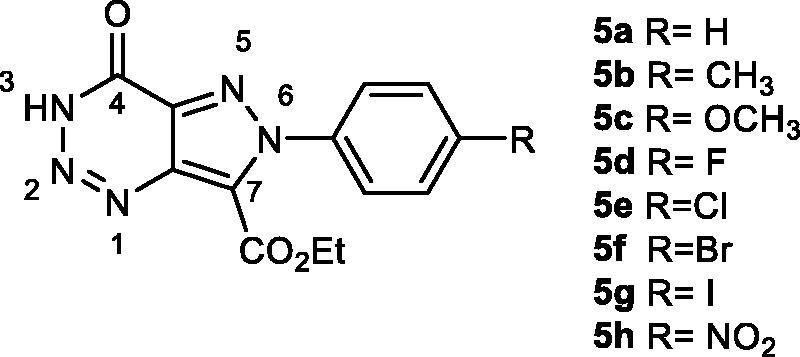
New design scaffold for potential GSK-3 inhibition.

The newly designed scaffold is shown in Figure 4. Docking studies were performed to evaluate the binding mode with the enzyme, varying the substituent in the *para* position of the phenyl group. The docking protocol previously described was used, and both, hydrophobic and electrostatic interactions were analysed. The substituents in the phenyl group were chosen considering commercially available starting materials.

**Figure 5.**
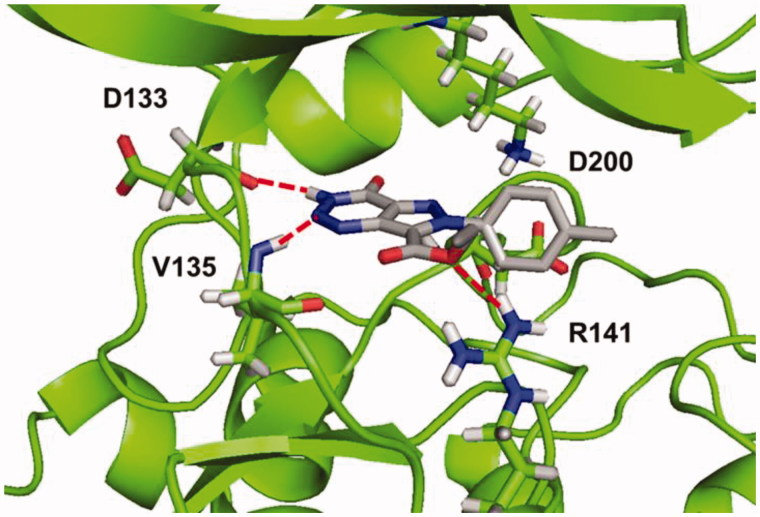
Binding mode for compound **5b** (R=CH_3_) in GSK-3 showing three relevant H-bonds with nearby residues.

As a result, all compounds showed a similar binding mode in the catalytic region of GSK-3, shown in Figure 5 for compound **5b** (R=CH_3_). Among the main interactions analysed, the two hydrogen bonds between triazine moiety and residues Asp133 and Val135 remain unaltered. Additionally, a new H-bond was formed between the carboethoxy group in C-7 and residue Arg141.

**Figure 6.**
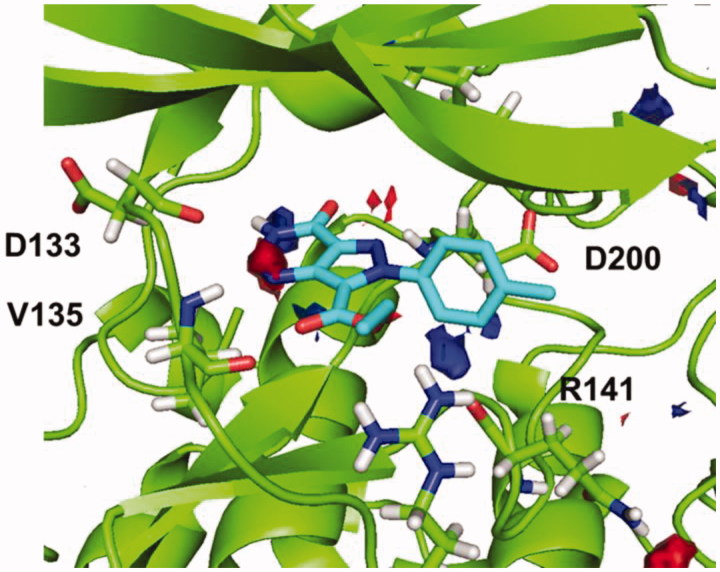
Superimposition of binding mode for compound **5b** (R=CH_3_) with polar regions predicted by the hotspot analysis in GSK-3 cavity.

Afterwards, docking results were combined with the hotspot analysis. The superimposition of the polar regions of the enzyme pocket and the docking pose obtained for compound **5b** are shown in Figure 6. The three H-bonds matches exactly with the hotspot prediction. Moreover, phenyl group was oriented towards the hydrophobic region and was able to occupy a larger area of the catalytic pocket. This analysis suggested that both, pyrazolotriazinone scaffold and the carboethoxy group in C-7, could be considered as chemical key features for the inhibition of GSK-3.

**Scheme 2.**
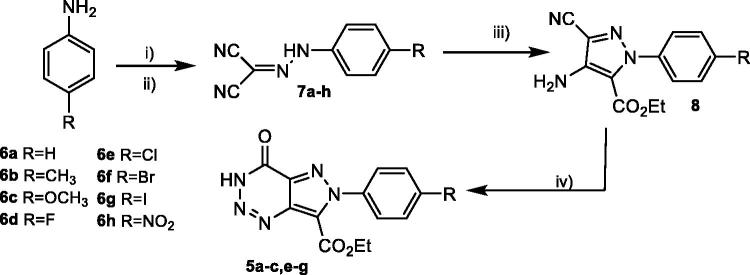
Synthesis of hydrazones **7a**–**h** and pyrazoles **8a**–**h**, precursor of the pyrazolotriazinones **5**, previously designed. (i) aq. NaNO_2_ [1.0 mmol], HCl [13.4 mmol] , 0 °C, 30 min; (ii) CH_2_(CN)_2_ [1.5 mmol], NaOAc.3H_2_O [31.0 mmol], H_2_O [8.5 ml], 0 °C, 2 h; (iii) Br-CH_2_-CO_2_Et [2.7 mmol], K_2_CO_3_ [1.0 mmol], DMF [3 ml], 90 °C, 5–7 h; (iv) NaNO_3_ [1.8 mmol], HCl:AcOH 3:1 [20 ml], 0–5 °C to r.t., 20 h.

With the aim to synthesise compounds ethyl 4-oxo-4,6-dihydro-3*H*-pyrazolo[4,3-*d*][1–3]triazine-7-carboxylates **5**, a multistep route was planned using *para*-substitutes anilines as starting reagents. Initially, the synthesis of *N*-aryl-hydrazones (**7a**–**h**) was carried out using commercially available anilines in a simple protocol previously described^33^. Sodium nitrite in aqueous hydrochloric acid was used for the diazotisation of anilines **6a**–**h**. Followed by the diazonium ion reacted with malononitrile to give the *N*-aryl-hydrazones (Scheme 2). In all cases, dicyano-hydrazones **7a**–**h** were obtained in excellent yields (Table 1).

**Table 1.** Synthesis of hydrazones **7a**–**h**, pyrazoles **8a**–**h** and pyrazolotriazinones **5a**–**c**, **e**–**g**.

R	Comp.	Yield^a^	Comp.	Yield	Comp.	Yield^b^
H	**7a**	99 %	**8a**	39 %	**5a**	35 %
CH_3_	**7b**	95 %	**8b**	38 %	**5b**	38 %
OCH_3_	**7c**	92 %	**8c**	42 %	**5c**	28 %
F	**7d**	86 %	**8d**	49 %	**5d**	–
Cl	**7e**	99 %	**8e**	52 %	**5e**	31 %
Br	**7f**	98 %	**8f**	40 %	**5f**	36 %
I	**7g**	94 %	**8g**	55 %	**5g**	33 %
NO_2_	**7h**	96 %	**8h**	18 %	**5h**	–

^a^Isolated product; ^b^Purified product.

Afterwards, the syntheses of ethyl 4-amino-3-cyano-1*H*-pyrazole-5-carboxylates **8a**–**h** were performed. As it is described in literature^33–36^, a methylene moiety is needed for the formation of pyrazole ring closure. In this approach, ethyl bromoacetate was used under basic conditions (K_2_CO_3_) in DMF at 90 °C for 5–7 h to afford the corresponding pyrazole (Scheme 2) with low to good yield (Table 1). Pyrazoles **8a** and **8b** are described in literature^35^, while compounds **8c**–**h** are all new compounds and were fully characterised in this work.

Finally, pyrazoles **8a**–**h** were subjected to diazotisation conditions to prepare novel 4-oxo-pyrazolo[4,3-*d*][1–3]triazine-7-carboxylates, using the protocol previously described for aminopyrazoles **3**^31^, as shown in Scheme 2.

**Figure 7.**
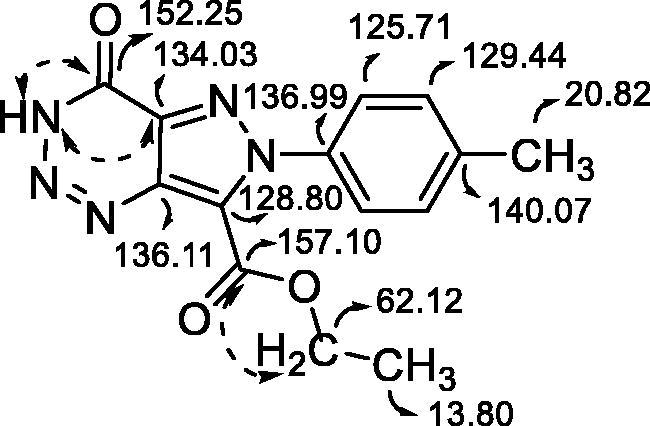
HMBC and ^13^C assignation of carbons (in ppm) and correlations for compound **5b**.

Yields of pyrazolotriazinones **5** (Table 1) were not as good as expected due to difficulties in the purification from the reaction mixture. In the case of diazotisation of compounds **8d** and **8h**, the corresponding pyrazolotriazines were not obtained, probably due to the electronegativity of the substituents in the phenyl ring that could destabilise or avoid the diazonium salt formation. It is known that the stability and reactivity of pyrazole-derived diazonium salts are strongly dependent on the nature of its substituents in N-1^37^. The structure of the 3*H*-pyrazolotriazinones tautomer was confirmed by NMR experiments agreeing with previous results^31^. As an example, the analysis of ^13 ^C and 2 D spectra for compound **5b** is shown in Figure 7. These experiments allowed the assignation of both carbonyl carbons, one from the triazine ring and the other from the carboethoxy group. Also, a correlation between H-3 and the carbonyl carbon from the triazine could be established that confirmed the presences of the tautomer *3H.*

Ethyl pyrazolotriazinones carboxylates **5a–c, e–g** were evaluated as potential GSK-3 inhibitors with the luminescent assay previously used^30^. As a result, derivative compound **5** acted as GSK-3 inhibitors at a fixed compound concentration of 10 μM. Thus, the concentration at which the 50% of inhibition (IC_50_) is produced was calculated for compounds **5a**–**c**, **e**–**g**, obtaining low micromolar-submicromolar values (Table 2).

**Table 2.** Biological inhibition of GSK-3 of ethyl 4-oxo-pyrazolo[4,3-*d*][1,2,3]triazine-7-carboxylates **5**.

R	Comp.	GSK-3 IC_50_ (μM)^a^
H	**5a**	4.0 ± 0.2
CH_3_	**5b**	0.9 ± 0.1
OCH_3_	**5c**	1.7 ± 0.3
Cl	**5e**	3.3 ± 0.3
Br	**5f**	1.4 ± 0.4
I	**5g**	0.8 ± 0.2

^a^IC_50_ values are reported as a mean value of three independent determinations.

**Figure 8.**
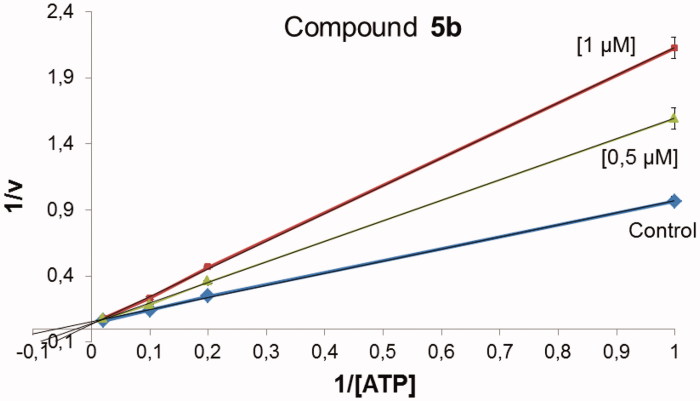
Kinetic data determined for **5b** suggesting an ATP-competitive behaviour.

To identify the type of inhibition of pyrazolotriazinones **5** on GSK-3, kinetic experiments were carried out. In that case, compound **5b** was evaluated at two different concentrations of 1 μM and 0.5 μM varying ATP concentration, while GS2 concentration remains constant. Comparable results were obtained for all compounds. As an example, the data obtained for compound **5b** is represented in a Lineweaver–Burk graph shown in Figure 8. This graphic suggests that pyrazolotriazinones acted as ATP-competitive inhibitors, indicating that they competed with the ATP in their binding mode to the enzyme.

The experimental data indicating that these compounds act as ATP-competitive inhibitors was consistent with docking studies, suggesting the binding mode in the catalytic region of GSK-3. In order to assess pyrazolotriazinones **5a-c**, **e-g** as potential new drug candidates for the treatment of Alzheimer’s disease, their ability to penetrate the blood–brain barrier (BBB) was evaluated. For this purpose, a permeability assay based on parallel artificial membrane was used (PAMPA methodology)^38^. This procedure is a high throughput technique that allows the prediction of permeability evaluation of organic compounds using a porcine brain lipid that emulates the blood–brain barrier.

The PAMPA assay was used to predict the *in vitro* permeability (*Pe*) of compounds **5a–c**, **e–g** and evaluate their brain penetration by passive diffusion. An assay validation was made comparing the reported permeability values of commercial drugs with the experimental data obtained employing this methodology. A good correlation between experimental-described values was obtained *Pe* (exp) = 0.7857(bibl) – 0.0816 (R^2^  =  0.972) (Figure 1SI). From this equation, and following the pattern established in the literature for BBB permeation prediction, we could classify compounds as CNS + when they present a permeability >3.1 × 10^−6 ^cm s^−1^. Considering these results, we can determine that compounds **5f** and **5g** would be able to cross the BBB by passive permeation (Table 3) while some others such as **5b–c** and **5e** are in the limit of a positive prediction.

**Table 3.** Permeability (Pe 10^−6 ^cm s^−1^) in the PAMPA-BBB assay for 10 commercial drugs (used in the experiment validation) and ethyl pyrazolo[4,3-*d*][1,2,3]triazine-7-carboxylates with their predictive penetration in the CNS.

Compound	*Pe* bibli. (10^−6^ cm s^−1^)^a^	*Pe* (10^−6^ cm s^−1^)^b^	BBB prediction
Atenolol	0.8	0.6 ± 0.4	–
Caffeine	1.3	1.1 ± 0.4	–
Desipramine	12	10.4 ± 0.1	–
Enoxacine	0.9	1.1 ± 0.6	–
Hydrocortisone	1.9	0.9 ± 0.3	–
Ofloxacine	0.8	0.5 ± 0.5	–
Piroxicam	2.5	0.5 ± 0.1	–
Promazine	8.8	8.4 ± 0.3	–
Testosterone	17	12.5 ± 2.5	–
Verapamil	16	11.9 ± 0.1	–
**5a**	–	1.1 ± 0.6	CNS−
**5b**	–	2.3 ± 0.3	CNS+/−
**5c**	–	1.6 ± 0.5	CNS+/−
**5e**	–	2.9 ± 0.7	CNS+/−
**5f**	–	3.20 ± 0.1	CNS+
**5g**	–	5.0 ± 1.0	CNS+

^a^Reference^38^; ^b^Data are the mean ± SD of three independent experiments.

Furthermore, to confirm cellular activity of our GSK-3 inhibitors, we check their anti-tau profile by exploring their rescue potential in the okadaic acid (OA)-induced neurodegeneration cell model, in comparison with the well know GSK-3 inhibitor named as TDZD8^50^. OA represents the most robust way to induce PHF-like tau hyperphosphorylation. Thus, OA-treated cell lines and primary neuronal cultures have been used as established cellular models of hyperphosphorylated tau-induced neurodegeneration^39^^,^^40^.

**Figure 9.**
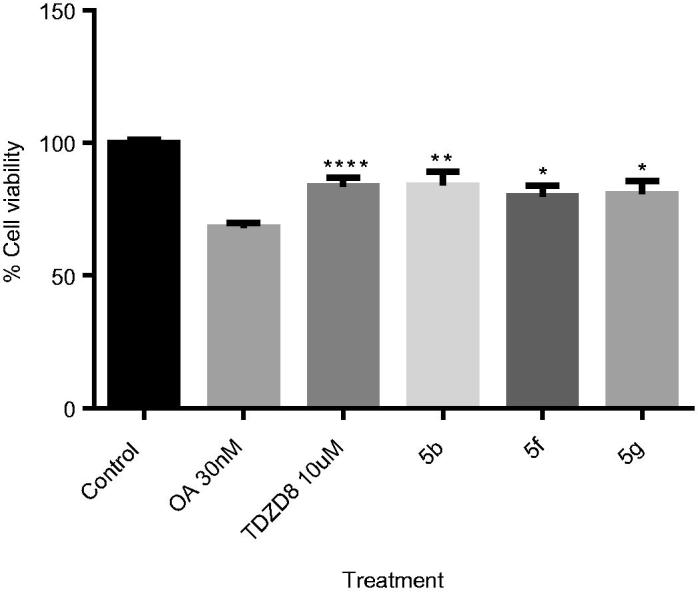
OA-induced neurodegeneration cell model. Effects of treatment with **5b, 5f** and **5e.** SH-SY5Y cells were treated with the GSK-3 inhibitors at 10 µM for 1 h and subsequently with OA (30 nM). The % of cell viability was measured by the MTT test. Results Mean ± SEM of three experiments, *p < .01, **p < .001, ***p < .0001, ****p < .00001.

We studied the effect of pyrazolotriazinones **5f**, **5 g**, and also **5b.** We select this last compound based on its submicromolar activity in GSK-3 together with the potential penetration in BBB. We used a fixed concentration of 10 µM in a human neuroblastoma cell line (*SH*-*SY5Y)* treated with OA at 30 nM (Figure 9). As expected, OA induced a decrease in cell viability higher than 70%. Importantly, the three pyrazolotriazinones assayed were able to increase the cell viability measured by the MTT test to the same level than the standard TDZD-8 does, probably due to the decrease on tau phosphorylation by GSK-3 inhibition.

Based on all results, ethyl 4-oxo-pirazolo[4,3-*d*][1–3]triazine-7-carboxylates **5f** and **5g**, which show low micromolar-submicromolar values of GSK-3 inhibition, were predicted to be able to pass the BBB, and show a neuroprotective profile against tau hyperphosphorylation in cell-based models could be considered as potential candidates for further studies for the treatment of AD.

## 4. Conclusions

In the first part of this study, pyrazolo[3,4-*d*][1–3]triazin-4-ones compounds (**4a**–**g**) were explored in their capacity to inhibit GSK-3. Although these compounds did not show inhibitory activity at a fixed concentration of 10 μM, docking studies were performed to determine their binding mode to the enzyme.

Secondly, based on the results obtained from computational studies such as docking and hotspot analysis, a new scaffold of pyrazolo[4,3-*d*][1–3]triazin-4-ones **5** was designed as potential GSK-3 inhibitors. Docking studies were performed to describe binding mode of **5** to the enzyme. Three hydrogen bonds were observed between each compound and residues Asp133, Val135, and Arg141. From these observations, the pyrazolotriazinone moiety and the carboethoxy group in C-7 were considered of significant importance for GSK-3 inhibition.

Finally, the designed 4-oxo-pyrazolo[4,3-*d*][1–3]triazine-7-carboxylates were prepared using a simple multistep protocol. These compounds showed *in vitro* GSK-3 inhibition in lower micro and submicromolar range, validating the computational calculations.

The ability of pyrazolotriazines to cross the BBB by passive diffusion was also evaluated using an *in vitro* model and the kinase activity was confirmed in a cell-based tau hyperphosphorylation model for the permeable compounds. As a result, those compounds that show great GSK-3 inhibition (**5f** and **5g**) were predicted to be able to penetrate the CNS showing neuroprotective activities against OA cell death.

Overall, new pharmacological tools to explore the role of GSK-3 in physiology and pathology were described. Moreover, some of the new brain permeable pyrazolotriazinones may be considered as new leads for further optimisation in the AD pharmacotherapy field.

## Supplementary Material

IENZ_1530223_Supplementary Material
