# Supplementary material for: Computer-aided molecular design of pyrazolotriazines targeting glycogen synthase kinase 3
Source: J Enzyme Inhib Med Chem. 2018 Oct 26;34(1):87–96. doi: 10.1080/14756366.2018.1530223 (PMC6211276; doi:10.1080/14756366.2018.1530223)
Supplement: IENZ_1530223_Supplementary Material [file IENZ_A_1530223_SM7835.docx]

**Computer-aided molecular design of pyrazolotriazines targeting glycogen synthase kinase 3**

M. Lourdes Sciú^a,b,d^, Victor Sebastián-Pérez^a,d^, Loreto Martinez-Gonzalez^a^, Rocio Benitez^a^, Daniel I. Perez^a^, Concepción Perez^c^, Nuria E. Campillo^a^, Ana Martinez*^a^ and E. Laura Moyano*^b^

^a^ Department of Chemical and Physical Biology, Centro de Investigaciones Biológicas (CIB, CSIC) Ramiro de Maeztu, 9, 28040 Madrid, Spain

^b^ INFIQC- Department of Organic Chemistry School of Chemical Sciences, National University of Córdoba, Haya de la Torre y Medina Allende, X5000HUA Córdoba, Argentine

^c^ Instituto de Química Médica (IQM, CSIC), Juan de la Cierva 3, 28006 Madrid, Spain

^d^ These authors contributed equally to this publication

**Table of contents**

Part 1. Supplementary figures

Part 2. Compound characterization data

**Part 1. Supplementary Figures**

**Figure 1S.** Linear correlation between experimental and reported permeability of commercial drugs using the PAMPA-BBB assay.

Part 2. Compound characterization data

ethyl 4-amino-3-cyano-1-phenyl-1*H*-pyrazole-5-carboxylate (**8a**):

^1^H spectrum

^1^H NMR (400 MHz, CD_3_CN, 25ºC): δ= 7.56 – 7.33 (m, 5H), 5.15 (s, 2H), 4.18 (q, *J* = 7.1 Hz, 2H), 1.11 (t, *J* = 7.1 Hz, 3H) ppm.

^13^C spectrum

^13^C NMR (101 MHz, ((CD_3_)_2_CO, 25ºC): δ= 159.7, 143.8, 141.5, 129.4 (C8, C10), 126.9 (C7, C11), 118.1, 114.7, 113.5, 61.5, 14.2 ppm.

HSQC-DEPT spectrum

HMBC spectrum

ethyl 4-amino-3-cyano-1-(*p*-tolyl)-1*H*-pyrazole-5-carboxylate (**8b**):

^1^H spectrum

^1^H NMR (400 MHz, (CD_3_)_2_CO, 25ºC): δ= 7.42 – 7.23 (m, 4H), 5.65 (s, 2H), 4.21 (q, *J* = 7.1 Hz, 2H), 2.41 (s, 3H), 1.14 (t, *J* = 7.1 Hz, 3H) ppm.

^13^C spectrum

^13^C NMR (101 MHz, CD_3_CN, 25ºC): δ= 159.9, 143.4, 140.5, 139.0, 131.1, 130.0 (C8, C10), 126.8 (C7, C11), 114.5, 113.8, 61.9, 21.23 14.3 ppm.

HSQC-DEPT spectrum

HMBC spectrum

ethyl 4-amino-3-cyano-1-(4-methoxyphenyl)-1*H*-pyrazole-5-carboxylate (**8c**):

^1^H spectrum

^1^H NMR (400 MHz, (CD_3_)_2_CO, 25ºC): δ= 7.45 – 7.37 (m, 2H), 7.07 – 7.01 (m, 2H), 5.62 (s, 2H), 4.21 (q, *J* = 7.1 Hz, 2H), 3.88 (s, 3H), 1.14 (t, *J* = 7.1 Hz, 3H) ppm.

^13^C spectrum

^13^C NMR (101 MHz, CD_3_CN, 25ºC): δ= 161.2, 159.9, 143.4, 134.5, 128.3 (C7, C11), 115.9, 114.6 (C8, C10), 114.6, 114.3, 113.9, 61.9, 56.4, 14.3 ppm

HSQC-DEPT spectrum

HMBC spectrum

IR (AgBr): υ= 3471, 3359 (NH), 2230 (CN), 1721 (C=O), 1617, 1514 (C=C and C=N ring) cm^-1^.

ethyl 4-amino-3-cyano-1-(4-fluorophenyl)-1*H*-pyrazole-5-carboxylate (**8d**):

^1^H spectrum

^1^H NMR (400 MHz, CDCl_3_, 25ºC): δ= 7.42 – 7.30 (m, 2H), 7.21 – 7.10 (m, 2H), 4.80 (s, 2H), 4.23 (q, *J* = 7.1 Hz, 2H), 1.17 (t, *J* = 7.1 Hz, 3H) ppm.

^13^C spectrum

^13^C NMR (101 MHz, (CD_3_)_2_CO), 25 ºC), δ= 163.6 (d, *J* = 246.9 Hz), 159.6, 143.7, 137.8, 129.2 (d, *J* = 9.1 Hz, C7, C11), 118.3, 116.1 (d, *J* = 23.4 Hz, C8, C10), 114.7, 113.4, 61.6, 14.2 ppm.

HSQC-DEPT spectrum

HMBC spectrum

IR (AgBr): υ= 3475, 3369 (NH), 2229 (CN), 1721 (C=O), 1616, 1511 (C=C and C=N ring) cm^-1^.

ethyl 4-amino-1-(4-chlorophenyl)-3-cyano-1*H*-pyrazole-5-carboxylate (**8e**):

^1^H spectrum

^1^H NMR (400 MHz, CD_3_CN, 25ºC): δ= 7.49 (d, *J* = 8.8 Hz, 2H), 7.41 (d, *J* = 8.8 Hz, 2H), 5.17 (s, 2H), 4.20 (q, *J* = 7.1 Hz, 2H), 1.14 (t, *J* = 7.1 Hz, 3H) ppm.

^13^C spectrum

^13^C NMR (101 MHz, CD_3_CN, 25 ºC): δ= 159.8, 143.6, 140.1, 135.5, 129.6 (C8, C10), 128.8, 128.6 (C7, C11), 115.1, 113.6, 62.1, 14.3 ppm

HSQC-DEPT spectrum

HMBC spectrum

IR (AgBr): υ= 3424, 3309 (NH), 2230 (CN), 1724 (C=O), 1627, 1498 (C=C and C=N ring) cm^-1^.

ethyl 4-amino-1-(4-bromophenyl)-3-cyano-1*H*-pyrazole-5-carboxylate (**8f**):

^1^H spectrum

^1^H NMR (400 MHz, (CDCl_3_, 25ºC): δ= 7.59 (d, *J* = 8.7 Hz, 2H), 7.27 (d, *J* = 8.7 Hz, 2H), 4.81 (s, 2H), 4.26 (q, *J* = 7.1 Hz, 2H), 1.20 (t, *J* = 7.1 Hz, 3H) ppm.

^13^C spectrum

^13^C NMR (101 MHz, CD_3_CN, 25ºC): δ= 159.8, 143.6, 140.6, 133.2, 132.7 (C8, C10), 128.9 (C7, C11), 123.5, 120.5, 113.6, 62.1, 14.3 ppm

HSQC-DEPT spectrum

HMBC spectrum

IR (AgBr): υ= 3426, 3309 (NH), 2230 (CN), 1723 (C=O), 1627, 1490 (C=C and C=N ring) cm^-1^.

ethyl 4-amino-3-cyano-1-(4-iodophenyl)-1*H*-pyrazole-5-carboxylate (**8g**):

^1^H spectrum

^1^H NMR (400 MHz, (CD_3_)_2_CO, 25ºC): δ= 7.95 – 7.86 (m, 2H), 7.37 – 7.31 (m, 2H), 5.70 (s, 2H), 4.24 (q, *J* = 7.1 Hz, 2H), 1.17 (t, *J* = 7.1 Hz, 3H) ppm.

^13^C spectrum

^13^C NMR (101 MHz, CD_3_CN, 25 ºC): δ= 159.8, 143.6, 141.2, 139.4, 138.7 (C8, C10), 128.9 (C7, C11), 115.2, 113.6, 95.0, 62.1, 14.3 ppm

HSQC-DEPT spectrum

HMBC spectrum

IR (AgBr): υ= 3450, 3361 (NH), 2233 (CN), 1700 (C=O), 1640, 1489 (C=C and C=N ring) cm^-1^.

ethyl 4-amino-3-cyano-1-(4-nitrophenyl)-1*H*-pyrazole-5-carboxylate (**8h**):

^1^H spectrum

^1^H NMR (400 MHz, (CD_3_)_2_SO, 25ºC): δ= 8.33 (d, *J* = 8.8 Hz, 2H), 7.81 (d, *J* = 8.8 Hz, 2H), 6.16 (s, 2H), 4.19 (q, *J* = 6.9 Hz, 2H), 1.13 (t, *J* = 7.1 Hz, 3H) ppm.

^13^C spectrum

^13^C NMR (101 MHz, CD_3_CN, 25 ºC): δ= 159.8, 148.8, 145.8, 143.9, 127.9 (C8, C10), 125.9, 125.0 (C7, C11), 116.3, 113.4, 62.3, 14.3 ppm.

HSQC-DEPT spectrum

HMBC spectrum

ethyl 4-oxo-6-phenyl-4,6-dihydro-3*H*-pyrazolo[4,3-*d*][1,2,3]triazine-7-carboxylate (**5a**):

^1^H spectrum

^1^H NMR (400 MHz, (CD_3_)_2_CO, 25ºC): δ= 13.90 (s, 1H), 7.77 – 7.54 (m, 5H), 4.38 (q, *J* = 7.1 Hz, 2H), 1.27 (t, *J* = 7.1 Hz, 3H) ppm.

13C spectrum

^13^C NMR (400 MHz, (CD_3_)_2_CO, 25ºC): δ= 158.2, 152.9, 140.8, 137.4, 135.6, 131.0, 130.6, 129.9 (C10, C12), 126.8 (C9, C13), 63.0, 14.2 ppm

HMBC spectrum

IR (AgBr): υ= 1723 (C=O), 1496 (C=N ring) cm^-1^.

ethyl 4-oxo-6-(*p*-tolyl)-4,6-dihydro-3*H*-pyrazolo[4,3-*d*][1,2,3]triazine-7-carboxylate (**5b**):

^1^H spectrum

^1^H NMR (400 MHz, (CD_3_)_2_SO, 25ºC): δ= 14.99 (s, 1H), 7.52 (d, *J* = 8.4 Hz, 2H), 7.39 (d, *J* = 8.1 Hz, 2H), 4.33 (q, *J* = 7.1 Hz, 2H), 2.43 (s, 3H), 1.21 (t, *J* = 7.1 Hz, 3H) ppm.

13C spectrum

^13^C NMR (400 MHz, (CD_3_)_2_SO, 25ºC): δ= 157.1, 152.3, 140.1, 137.0, 136.1, 134.0, 129.4 (C10, C12), 128.8, 125.7 (C9, C13), 62.1, 20.8, 13.8 ppm

COSY spectrum

HSQC-DEPT spectrum

HMBC spectrum

IR (AgBr): υ= 1706, 1725 (C=O), 1511 (C=N ring) cm^-1^.

ethyl 6-(4-methoxyphenyl)-4-oxo-4,6-dihydro-3*H*-pyrazolo[4,3*-d*][1,2,3]triazine-7-carboxylate (**5c**):

^1^H spectrum

^1^H NMR (400 MHz, CDCl_3_, 25ºC): δ= 11.55 (s, 1H), 7.47 (d, *J* = 8.9 Hz, 2H), 7.03 (d, *J* = 8.9 Hz, 2H), 4.47 (q, *J* = 7.1 Hz, 2H), 3.90 (s, 3H), 1.37 (t, *J* = 7.1 Hz, 3H) ppm.

^13^C spectrum

^13^C NMR (101 MHz, CDCl_3_, 25ºC): δ= 161.2, 158.8, 157.7, 151.9, 134.4, 132.2, 127.1 (C9, C13), 114.3 (C10, C12), 109.9, 63.0, 55.8, 29.9 ppm.

HSQC-DEPT spectrum

HMBC spectrum

IR (AgBr): υ= 1714, 1725 (C=O), 1512 (C=N ring) cm^-1^.

ethyl 6-(4-chlorophenyl)-4-oxo-4,6-dihydro-3*H*-pyrazolo[4,3-*d*][1,2,3]triazine-7-carboxylate (**5e**):

^1^H spectrum

^1^H NMR (400 MHz, CD_3_CN, 25ºC): δ= 12.70 (s, 1H), 7.59 (m, 4H), 4.38 (q, *J* = 7.1 Hz, 2H), 1.26 (t, *J* = 7.1 Hz, 3H) ppm.

^13^C spectrum

^13^C NMR (101 MHz, CD_3_CN, 25ºC): δ= 158.3, 153.1, 139.4, 137.6, 136.7, 135.8, 130.8, 130.1 (C10, C12), 128.7 (C9, C13), 63.6, 14.2 ppm.

HMBC spectrum

IR (AgBr): υ= 1723, 1710 (C=O), 1496 (C=N ring) cm^-1^.

ethyl 6-(4-bromophenyl)-4-oxo-4,6-dihydro-3*H*-pyrazolo[4,3-*d*][1,2,3]triazine-7-carboxylate (**5f**):

^1^H spectrum

^1^H NMR (400 MHz, (CD_3_)_2_CO, 25ºC): δ= 13.93 (s, 1H), 7.83 (d, *J* = 8.8 Hz, 2H), 7.67 (d, *J* = 8.8 Hz, 2H), 4.41 (q, *J* = 7.1 Hz, 2H), 1.30 (t, *J* = 7.1 Hz, 3H) ppm.

^13^C spectrum

^13^C NMR (101 MHz, (CD_3_)_2_CO, 25ºC): δ= 158.2, 152.8, 140.0, 137.4, 135.8, 133.0 (C10, C12), 130.6, 129.0 (C9, C13), 124.6, 63.1, 14.3 ppm.

COSY spectrum

HSQC-DEPT spectrum

HMBC spectrum

IR (AgBr): υ= 1710 broad signal (C=O), 1490 (C=N ring) cm^-1^.

ethyl 6-(4-iodophenyl)-4-oxo-4,6-dihydro-3*H*-pyrazolo[4,3-*d*][1,2,3]triazine-7-carboxylate (**5g**):

1H spectrum

^1^H NMR (400 MHz, (CD_3_)_2_CO, 25ºC): δ= 13.90 (s, 1H), 7.77 – 7.54 (m, 4H), 4.38 (q, *J* = 7.1 Hz, 2H), 1.27 (t, *J* = 7.1 Hz, 3H) ppm.

13C spectrum

^13^C NMR (101 MHz, (CD_3_)_2_CO, 25ºC): δ= 158.2, 152.8, 140.7, 139.1 (C10, C12), 137.4, 135.8, 130.6, 128.9 (C9, C13), 96.3, 63.1, 14.3 ppm

COSY spectrum

HSQC-DEPT spectrum

HMBC spectrum

IR (AgBr): υ= 1723 broad signal (C=O), 1490 (C=N ring) cm^-1^.
